# *cyclo*-Tetra­kis(μ_2_-d-penicillaminato-κ^4^ *N*,*S*:*O*,*S*)tetra­palladium(II) 9.75-hydrate

**DOI:** 10.1107/S160053681301088X

**Published:** 2013-04-27

**Authors:** Anzu Yokoi, Nobuto Yoshinari, Takumi Konno

**Affiliations:** a Toyonaka, Osaka 560-0043, Department of Chemistry, Graduate School of Science, Osaka University, Japan

## Abstract

The asymmetric unit of the title compound, [Pd_4_(C_5_H_9_NO_2_S)_4_]·9.75H_2_O, contains two neutral tetranuclear complex molecules with similar conformations and 19.5 solvent water mol­ecules. Of the 21 independent water molecules, three exhibit an occupancy of one-half. In each tetranuclear complex molecule, the four Pd^II^ atoms have a square-planar coordination environment and are spanned by four d-penicillaminate ligands in a κ^4^ *N*,*S*:*S*,*O* coordination mode, forming an eight-membered Pd_4_S_4_ metallacycle. In the crystal, two tetra­nuclear mol­ecules are connected to each other through eight N—H⋯O hydrogen bonds between amine and carboxyl­ate groups, constructing a cylindrical dimer. The dimers are further hydrogen-bonded with the solvent water mol­ecules, completing a three-dimensional network.

## Related literature

For background to this class of compound, see: Igashira-Kamiyama & Konno (2011 ▶). For related structures, see: Yoshinari *et al.* (2009 ▶).
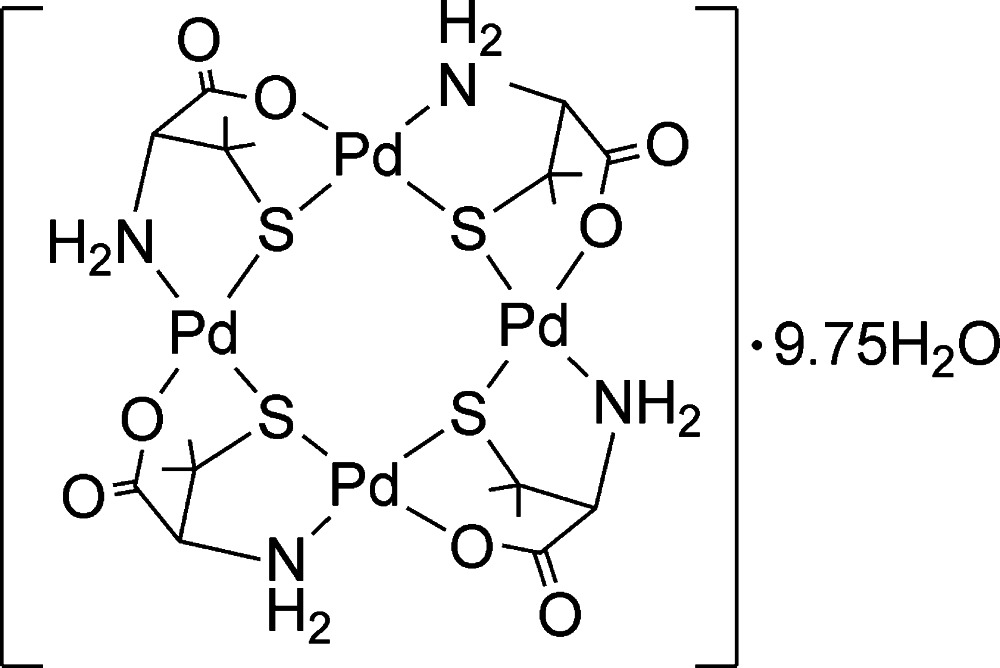


## Experimental

### 

#### Crystal data

[Pd_4_(C_5_H_9_NO_2_S)_4_]·9.75H_2_O
*M* *_r_* = 1190.03
Triclinic, 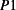

*a* = 12.4517 (3) Å
*b* = 13.2680 (4) Å
*c* = 15.1666 (11) Å
α = 113.527 (8)°
β = 98.425 (7)°
γ = 108.557 (8)°
*V* = 2067.3 (3) Å^3^
*Z* = 2
Mo *K*α radiation
μ = 1.98 mm^−1^
*T* = 200 K
0.25 × 0.10 × 0.03 mm

#### Data collection

Rigaku R-AXIS RAPID diffractometer
Absorption correction: multi-scan (*ABSCOR*; Rigaku, 1995 ▶) *T*_min_ = 0.730, *T*_max_ = 0.916
20447 measured reflections
15410 independent reflections
15064 reflections with *I* > 2σ(*I*)
*R*_int_ = 0.027

#### Refinement

*R*[*F* ^2^ > 2σ(*F* ^2^)] = 0.025
*wR*(*F* ^2^) = 0.062
*S* = 1.08
15410 reflections
1052 parameters
67 restraints
H atoms treated by a mixture of independent and constrained refinement
Δρ_max_ = 1.52 e Å^−3^
Δρ_min_ = −0.68 e Å^−3^
Absolute structure: Flack (1983 ▶), 6026 Friedel pairs
Flack parameter: −0.024 (13)

### 

Data collection: *PROCESS-AUTO* (Rigaku, 2000 ▶); cell refinement: *PROCESS-AUTO*; data reduction: *PROCESS-AUTO*; program(s) used to solve structure: *SHELXS97* (Sheldrick, 2008 ▶); program(s) used to refine structure: *SHELXL97* (Sheldrick, 2008 ▶); molecular graphics: *Yadokari-XG 2009* (Kabuto *et al.*, 2009 ▶); software used to prepare material for publication: *Yadokari-XG 2009*.

## Supplementary Material

Crystal structure: contains datablock(s) I, global. DOI: 10.1107/S160053681301088X/is5266sup1.cif

Structure factors: contains datablock(s) I. DOI: 10.1107/S160053681301088X/is5266Isup2.hkl

Supplementary material file. DOI: 10.1107/S160053681301088X/is5266Isup4.mol

Additional supplementary materials:  crystallographic information; 3D view; checkCIF report

## Figures and Tables

**Table 1:** Hydrogen-bond geometry (Å, °)

*D*—H⋯*A*	*D*—H	H⋯*A*	*D*⋯*A*	*D*—H⋯*A*
N1—H1⋯O26^i^	0.92	2.06	2.963 (5)	169
N1—H1*A*⋯O15^ii^	0.92	2.21	3.109 (4)	166
N2—H2⋯O27	0.92	2.35	3.139 (5)	143
N2—H2*A*⋯O13^ii^	0.92	2.11	2.928 (5)	148
N3—H3⋯O35*B*	0.92	2.01	2.889 (8)	160
N3—H3⋯O36*A*	0.92	2.23	3.045 (8)	147
N3—H3*A*⋯O11^ii^	0.92	2.37	3.278 (5)	167
N4—H4⋯O29^iii^	0.92	1.98	2.886 (5)	170
N4—H4*A*⋯O9^ii^	0.92	2.04	2.917 (4)	160
N5—H5⋯O18^i^	0.92	2.09	3.008 (5)	172
N5—H5*A*⋯O7^iv^	0.92	2.19	3.094 (4)	167
N6—H6⋯O27^v^	0.92	2.44	3.183 (5)	138
N6—H6⋯O28	0.92	2.48	3.326 (5)	153
N6—H6*A*⋯O5^iv^	0.92	2.21	3.041 (5)	151
N7—H7⋯O23	0.92	2.03	2.938 (5)	170
N7—H7*A*⋯O3^iv^	0.92	2.16	3.066 (5)	169
N8—H8⋯O28^vi^	0.92	1.98	2.896 (5)	171
N8—H8*A*⋯O1^iv^	0.92	2.06	2.930 (4)	157
O17—H17*F*⋯O26^iv^	0.85 (2)	1.96 (2)	2.798 (5)	167 (6)
O17—H17*G*⋯O16^vii^	0.86 (2)	1.93 (2)	2.775 (4)	170 (5)
O18—H18*F*⋯O17	0.84 (2)	2.00 (3)	2.818 (5)	165 (6)
O18—H18*G*⋯O20	0.85 (2)	1.98 (2)	2.814 (5)	165 (5)
O19—H19*F*⋯O8^vii^	0.85 (2)	2.05 (2)	2.892 (5)	168 (5)
O19—H19*G*⋯O2^viii^	0.85 (2)	1.94 (3)	2.760 (5)	160 (6)
O20—H20*F*⋯O21	0.85 (2)	1.88 (2)	2.709 (5)	167 (6)
O20—H20*G*⋯O8^ix^	0.86 (2)	1.94 (3)	2.742 (4)	156 (5)
O21—H21*F*⋯O22	0.86 (2)	1.97 (3)	2.805 (6)	162 (5)
O21—H21*G*⋯O30	0.87 (2)	2.00 (2)	2.857 (6)	169 (6)
O22—H22*F*⋯O10^vii^	0.85 (2)	1.95 (2)	2.802 (5)	174 (6)
O22—H22*G*⋯O16^viii^	0.86 (2)	2.21 (4)	2.915 (5)	139 (6)
O23—H23*F*⋯O20	0.85 (2)	1.96 (2)	2.796 (5)	168 (5)
O23—H23*G*⋯O25^iv^	0.85 (2)	1.96 (2)	2.782 (5)	162 (5)
O24—H24*F*⋯O19^vi^	0.85 (2)	1.97 (2)	2.814 (5)	177 (6)
O24—H24*G*⋯O32	0.85 (2)	1.90 (2)	2.736 (6)	168 (6)
O25—H25*F*⋯O24^iii^	0.87 (2)	1.91 (2)	2.770 (6)	170 (6)
O25—H25*G*⋯O12^ii^	0.85 (2)	1.97 (3)	2.788 (5)	159 (5)
O26—H26*F*⋯O23^ii^	0.86 (2)	1.95 (2)	2.791 (5)	167 (6)
O26—H26*G*⋯O31	0.85 (2)	1.95 (2)	2.788 (6)	169 (6)
O27—H27*F*⋯O6^vi^	0.85 (2)	1.88 (3)	2.687 (5)	158 (5)
O27—H27*G*⋯O14^ii^	0.84 (2)	1.81 (2)	2.650 (5)	175 (5)
O28—H28*F*⋯O10	0.82 (2)	2.02 (2)	2.810 (5)	161 (5)
O28—H28*G*⋯O27^v^	0.82 (2)	2.00 (3)	2.797 (5)	164 (5)
O29—H29*F*⋯O2	0.87 (2)	1.98 (2)	2.834 (5)	168 (6)
O29—H29*G*⋯O27	0.85 (2)	1.98 (2)	2.802 (5)	160 (5)
O30—H30*F*⋯O12	0.86 (2)	2.28 (5)	2.911 (7)	130 (6)
O30—H30*G*⋯O19^iv^	0.86 (2)	1.95 (3)	2.791 (5)	165 (6)
O31—H31*F*⋯O33^ii^	0.84 (2)	2.17 (3)	2.946 (8)	154 (7)
O31—H31*G*⋯O4	0.86 (2)	2.08 (2)	2.902 (6)	161 (5)
O32—H32*F*⋯O34	0.92 (2)	2.07 (2)	2.823 (7)	139 (3)
O32—H32*G*⋯O31	0.86 (2)	1.98 (2)	2.837 (7)	178 (9)
O33—H33*F*⋯O17	0.88 (2)	2.11 (4)	2.877 (6)	146 (7)
O33—H33*G*⋯O30^vi^	0.87 (2)	1.90 (2)	2.766 (6)	170 (7)
O34—H34*F*⋯O18	0.85 (2)	2.12 (4)	2.906 (6)	154 (8)
O34—H34*G*⋯O6^vi^	0.86 (2)	2.02 (3)	2.815 (6)	154 (6)
O35*A*—H35*F*⋯O25	0.85 (2)	2.14 (3)	2.986 (9)	174 (13)
O35*A*—H35*G*⋯O4	0.85 (2)	2.05 (5)	2.848 (9)	157 (12)
O35*B*—H35*H*⋯O4	0.83 (2)	2.45 (8)	3.135 (9)	141 (10)
O35*B*—H35*I*⋯O25	0.84 (2)	2.03 (5)	2.802 (10)	152 (9)
O36*A*—H36*F*⋯O22	0.85 (2)	2.14 (5)	2.949 (9)	159 (12)
O36*A*—H36*G*⋯O35*A*	0.85 (2)	1.93 (5)	2.746 (12)	158 (12)

Symmetry codes: (i) 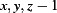; (ii) 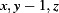; (iii) 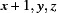; (iv) 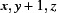; (v) 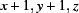; (vi) 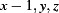; (vii) 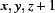; (viii) 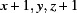; (ix) 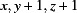.

**Fig. 1.**
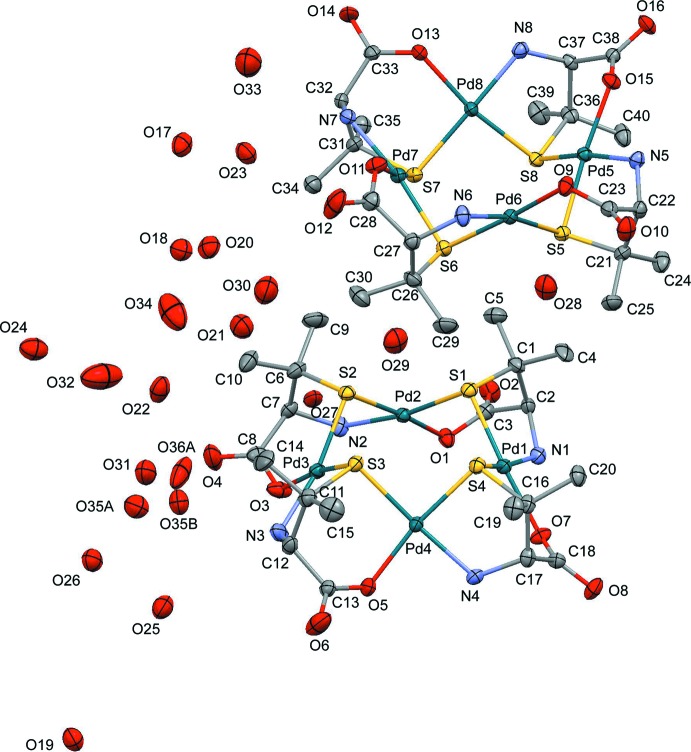
A perspective view of the title compound, showing the atom-numbering scheme. Displacement ellipsoids are drawn at the 50% probability level and H atoms have been omitted for clarity.

**Fig. 2.**
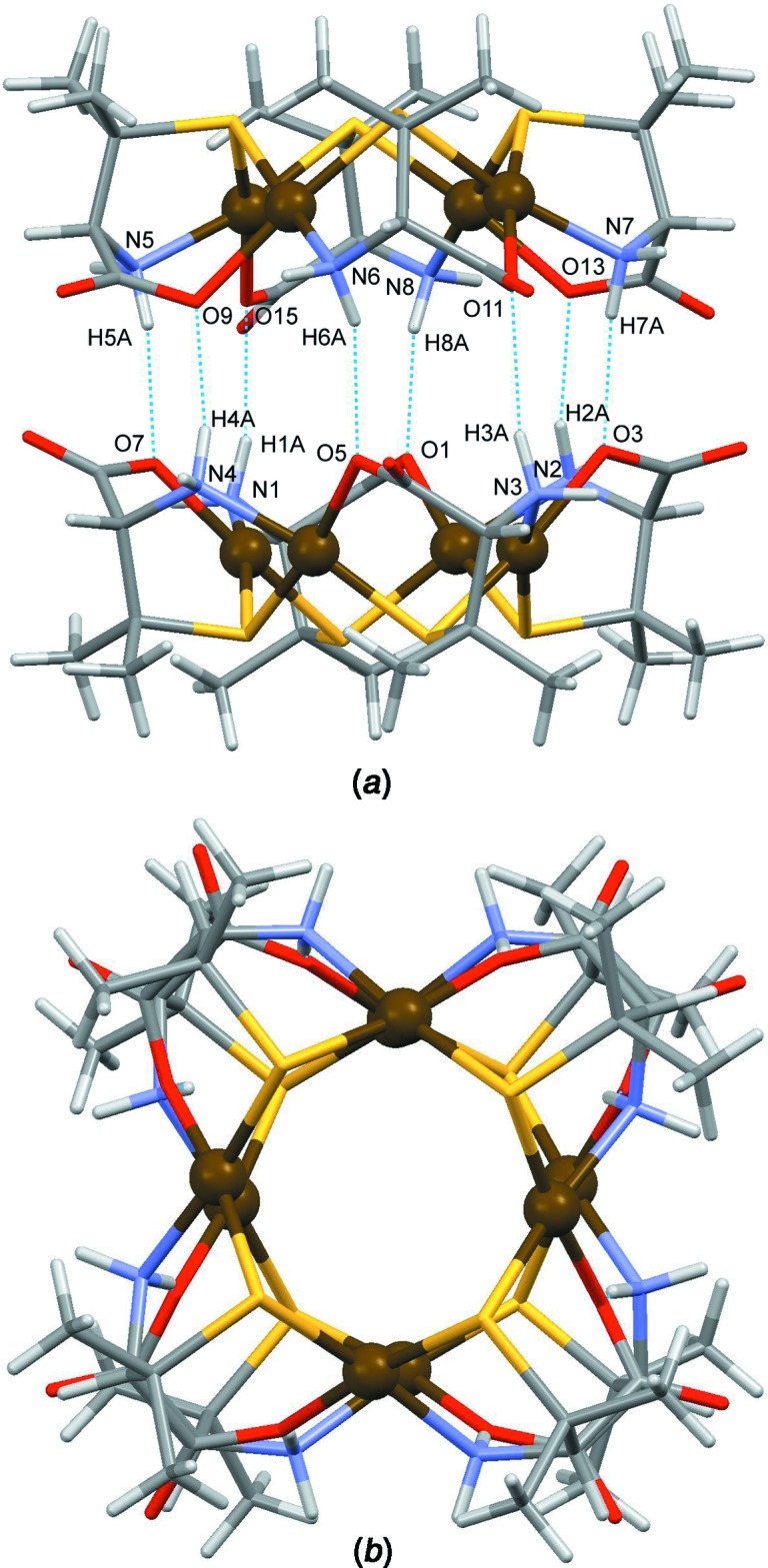
(a) Side and (b) top views of the cylindrical dimer structure in the title compound. H atoms have been omitted for clarity. Dashed lines indicate intermolecular N—H···O hydrogen bonds.

## supplementary crystallographic information

### Comment

*D*-Penicillamine (C_5_H_11_NSO_2_, D—H_2_pen), which is a sulfur-containing amino acid, has been known as a unique multidentate chiral ligand that has three kinds of coordination sites, amine N, carboxylate O, and thiolate S atoms (Igashira-Kamiyama & Konno, 2011). Recently, we found that an S-bridged cyclic tetranuclear Pd^II^ complex with D-pen, [Pd_4_Cl_4_(D-Hpen)_4_].7H_2_O, gradually converts to an S-bridged cyclic trinuclear Pd^II^ structure in [Pd_3_(D-pen)_3_].4.5H_2_O (II), by treating with K_2_CO_3_ in water (Yoshinari *et al.*, 2009). We assumed that some intermediate species exists in this reaction because not only the nuclearity of the complex (Pd^II^_4_*versus*. Pd^II^_3_) but also the coordination mode of D-pen (*κ*^2^*N*,*S*:*κS versus. κ*^2^*N*,*S*:*κ*^2^*S*,*O*) are required to be changed for this structural conversion. In the course of our trials to find an intermediate species, we were able to isolate crystals (I), the composition of which is different from those of [Pd_4_Cl_4_(D-Hpen)_4_].7H_2_O and [Pd_3_(D-pen)_3_].4.5H_2_O, and its structure was determined by single-crystal X-ray crystallography. Herein we report that crystal (I) thus obtained has a new cyclic tetranuclear Pd^II^ structure in [Pd^II^_4_(D-pen)_4_].9.75H_2_O, which forms an intriguing supramolecular dimer structure.

The asymmetric unit of (I) contains two tetranuclear Pd^II^ molecules, [Pd_4_(D-pen)_4_], and nineteen and a half solvated water molecules (Figure 1). The two tetranuclear molecules are essentially the same as each other, comprising of four square-planar Pd^II^ atoms and four D-pen ligands. Each D-pen ligand bridges two Pd^II^ centers in a *κ*^2^*N*,*S*:*κ*^2^*S*,*O* coordination mode, while each Pd^II^ atom is coordinated by two D-pen ligands in *N*,*S*- and *S*,*O*- chelating modes, forming a crown-type eight-membered Pd_4_S_4_ metallacycle. Note that amine and carboxy groups cover one face of the metallacycle, while thiolate and methyl groups cover another face in (I). It has been shown that similar hydrophilic and hydrophobic surfaces are formed in the S-bridged metallacycle structure in (II).

The Pd—S distances and S—Pd—S angles in (I) [Pd—S = 2.2261 (11)–2.2968 (10) Å, S—Pd—S = 89.76 (4)–93.65 (4)°] are similar to those in (II) [Pd—S = 2.2427 (15)–2.2934 (15) Å, S—Pd—S = 90.08 (6)–92.66 (6)°]. However, the Pd—S—Pd angles in (I) [96.90 (4)–102.42 (4)°] are closer to the ideal tetrahedral angle of 109.5° compared with those in (II) [Pd—S—Pd = 82.89 (5)–91.65 (6)°], while the *trans* angles around Pd^II^ atoms in (I) [167.45 (12)–172.13 (8)°] are slightly smaller than those in (II) [170.41 (13)–176.67 (16)°]. This implies that the distortion around S atoms is larger but the distortion around Pd atoms is smaller in (I), compared with those in (II).

The most remarkable structural feature in (I) is the construction of a cylindrical supramolecular structure, in which two cyclic tetranuclear molecules face to each other to form eight intermolecular N—H···O hydrogen bonds [N···O = 2.917 (4)–3.278 (5) Å] (Figure 2). None of solvent molecules are accommodated in the cylinder despite the presence of a void space with a diameter of *ca* 4 Å. All of the water molecules are participated in the formation of O—H···O and/or N—H···O hydrogen bonds with neighboring water molecules and/or tetranuclear molecules [O···O = 2.650 (5)–3.135 (9) Å, N···O = 2.886 (5)–3.326 (5) Å], completing three dimensional structure in (I) (Table 1).

### Experimental

To an orange suspension containing 100 mg (0.08 mmol) of [Pd_4_(D-Hpen)_4_Cl_4_].7H_2_O in water (10 ml) was added an aqueous K_2_CO_3_ (0.2 *M*, 1 ml). From the resulting orange solution, a yellow crystalline powder of (I) was immediately precipitated, which was collected by filtration. Yield: 43 mg (46%). ^1^H NMR in DMSO-*d*_6_ (500 MHz): *δ* 5.84 (dd, *J* = 11.1, 7.5 Hz, 1H, NH_2_), 4.95 (d, *J* = 11.2 Hz, 1H, NH_2_), 3.15 (d, J = 7.6 Hz, 1H, CH), 1.64 (s, 3H, CH_3_), 1.64 (s, 3H, CH_3_). IR (KBr): 1610 cm^-1^ (ν_CO_ for COO^-^). Single-crystals of (I) suitable for X-ray analysis were obtained by recrystallization of the powder from a small amount of ethanol/water (1:1).

### Refinement

H atoms bound to C and N atoms were placed at calculated positions [C—H = 0.98 (CH_3_), 0.99 (CH_2_) or 1.00 Å (CH) and N—H = 0.92 Å (NH_2_)] and refined as riding with *U*_iso_(H) = 1.2*U*_eq_(C,N) for CH and NH_2_, and 1.5*U*_eq_(C) for CH_3_. One and a half water molecules were disordered, and these molecules were refined with occupancies of 0.5. H atoms of water molecules were placed so as to form reasonable hydrogen bonding. All H atoms bound to O atoms were refined with restrained geometric and displacement parameters [O—H = 0.85 (2) Å, H···H = 1.38 (2) Å and *U*_iso_(H) = 1.2*U*_eq_(O)]. In addition, one H atom (H32F) was refined with an additional intermolecular H···O distance restraint so as to form reasonable hydrogen bonding. Reflections of (-6 14 -9), (-13 2 1), (-12 -3 0) and (-12 2 14) were removed to improve the data quality.

### Crystal data

**Table:**

[Pd_4_(C_5_H_9_NO_2_S)_4_]·9.75H_2_O	*Z* = 2
*M**_r_* = 1190.03	*F*(000) = 1187
Triclinic, *P*1	*D*_x_ = 1.912 Mg m^−^^3^
Hall symbol: P 1	Mo *K*α radiation, λ = 0.71075 Å
*a* = 12.4517 (3) Å	Cell parameters from 20017 reflections
*b* = 13.2680 (4) Å	θ = 3.1–27.5°
*c* = 15.1666 (11) Å	µ = 1.98 mm^−^^1^
α = 113.527 (8)°	*T* = 200 K
β = 98.425 (7)°	Platelet, yellow
γ = 108.557 (8)°	0.25 × 0.10 × 0.03 mm
*V* = 2067.3 (3) Å^3^	

### Data collection

**Table:**

Rigaku R-AXIS RAPID diffractometer	15410 independent reflections
Radiation source: fine-focus sealed tube	15064 reflections with *I* > 2σ(*I*)
Graphite monochromator	*R*_int_ = 0.027
Detector resolution: 10.000 pixels mm^-1^	θ_max_ = 27.5°, θ_min_ = 3.1°
ω scans	*h* = −16→14
Absorption correction: multi-scan (*ABSCOR*; Rigaku, 1995)	*k* = −16→17
*T*_min_ = 0.730, *T*_max_ = 0.916	*l* = −19→19
20447 measured reflections	

### Refinement

**Table:**

Refinement on *F*^2^	Secondary atom site location: difference Fourier map
Least-squares matrix: full	Hydrogen site location: inferred from neighbouring sites
*R*[*F*^2^ > 2σ(*F*^2^)] = 0.025	H atoms treated by a mixture of independent and constrained refinement
*wR*(*F*^2^) = 0.062	*w* = 1/[σ^2^(*F*_o_^2^) + (0.0317*P*)^2^ + 0.9989*P*] where *P* = (*F*_o_^2^ + 2*F*_c_^2^)/3
*S* = 1.08	(Δ/σ)_max_ = 0.001
15410 reflections	Δρ_max_ = 1.52 e Å^−^^3^
1052 parameters	Δρ_min_ = −0.68 e Å^−^^3^
67 restraints	Absolute structure: Flack (1983), 6026 Friedel pairs
Primary atom site location: structure-invariant direct methods	Flack parameter: −0.024 (13)

### Special details

**Table:**

Geometry. All e.s.d.'s (except the e.s.d. in the dihedral angle between two l.s. planes) are estimated using the full covariance matrix. The cell e.s.d.'s are taken into account individually in the estimation of e.s.d.'s in distances, angles and torsion angles; correlations between e.s.d.'s in cell parameters are only used when they are defined by crystal symmetry. An approximate (isotropic) treatment of cell e.s.d.'s is used for estimating e.s.d.'s involving l.s. planes.
Refinement. Refinement of *F*^2^ against ALL reflections. The weighted *R*-factor *wR* and goodness of fit *S* are based on *F*^2^, conventional *R*-factors *R* are based on *F*, with *F* set to zero for negative *F*^2^. The threshold expression of *F*^2^ > σ(*F*^2^) is used only for calculating *R*-factors(gt) *etc*. and is not relevant to the choice of reflections for refinement. *R*-factors based on *F*^2^ are statistically about twice as large as those based on *F*, and *R*- factors based on ALL data will be even larger.

### Fractional atomic coordinates and isotropic or equivalent isotropic displacement parameters (Å^2^)

**Table:**

	*x*	*y*	*z*	*U*_iso_*/*U*_eq_	Occ. (<1)
Pd1	0.56254 (2)	0.15412 (2)	−0.01146 (2)	0.01755 (6)	
Pd2	0.41117 (2)	0.20457 (2)	0.15893 (2)	0.01745 (6)	
Pd3	0.65618 (3)	0.29031 (2)	0.35747 (2)	0.01965 (7)	
Pd4	0.80963 (2)	0.23751 (2)	0.19018 (2)	0.01745 (6)	
Pd5	0.38317 (2)	0.68823 (2)	−0.09373 (2)	0.01653 (6)	
Pd6	0.64056 (2)	0.76363 (2)	0.08388 (2)	0.01663 (6)	
Pd7	0.52043 (2)	0.81899 (2)	0.27928 (2)	0.01811 (6)	
Pd8	0.26082 (2)	0.75142 (2)	0.10469 (2)	0.01650 (6)	
S1	0.48748 (9)	0.28612 (8)	0.06247 (7)	0.02000 (19)	
S2	0.53843 (9)	0.36787 (8)	0.30163 (7)	0.01988 (19)	
S3	0.80787 (9)	0.39725 (8)	0.32436 (7)	0.02019 (19)	
S4	0.75113 (9)	0.30047 (8)	0.08309 (7)	0.01972 (19)	
S5	0.49291 (8)	0.59918 (8)	−0.05455 (7)	0.01876 (19)	
S6	0.58784 (9)	0.68416 (9)	0.18395 (7)	0.01953 (19)	
S7	0.33138 (8)	0.67908 (8)	0.20419 (7)	0.01933 (19)	
S8	0.23872 (8)	0.59553 (8)	−0.04008 (7)	0.01813 (18)	
O1	0.2813 (2)	0.0429 (2)	0.0384 (2)	0.0231 (6)	
O2	0.1159 (3)	0.0122 (3)	−0.0646 (2)	0.0332 (7)	
O3	0.5311 (3)	0.1763 (3)	0.3917 (3)	0.0332 (7)	
O4	0.4366 (4)	0.2102 (4)	0.5046 (3)	0.0484 (10)	
O5	0.8676 (3)	0.1593 (3)	0.2715 (2)	0.0272 (6)	
O6	1.0310 (3)	0.2146 (4)	0.3895 (3)	0.0510 (11)	
O7	0.6082 (2)	0.0152 (2)	−0.0941 (2)	0.0231 (6)	
O8	0.7179 (3)	−0.0477 (3)	−0.1856 (2)	0.0299 (7)	
O9	0.7102 (3)	0.8570 (2)	0.0075 (2)	0.0238 (6)	
O10	0.8157 (3)	0.8105 (3)	−0.0954 (2)	0.0333 (7)	
O11	0.6842 (3)	0.9670 (3)	0.3624 (2)	0.0282 (7)	
O12	0.8575 (3)	1.0212 (4)	0.4671 (3)	0.0553 (11)	
O13	0.2651 (3)	0.9032 (3)	0.2224 (2)	0.0260 (6)	
O14	0.2040 (3)	0.9751 (3)	0.3530 (3)	0.0410 (9)	
O15	0.2990 (2)	0.7811 (3)	−0.1378 (2)	0.0229 (6)	
O16	0.1355 (3)	0.7755 (3)	−0.2233 (2)	0.0275 (6)	
N1	0.3900 (3)	0.0411 (3)	−0.1125 (2)	0.0203 (7)	
H1	0.3935	0.0211	−0.1772	0.024*	
H1A	0.3649	−0.0292	−0.1079	0.024*	
N2	0.3325 (3)	0.1506 (3)	0.2542 (3)	0.0240 (7)	
H2	0.2517	0.1275	0.2301	0.029*	
H2A	0.3433	0.0831	0.2484	0.029*	
N3	0.7798 (3)	0.2570 (4)	0.4357 (3)	0.0296 (8)	
H3	0.7763	0.2823	0.5008	0.036*	
H3A	0.7565	0.1748	0.4061	0.036*	
N4	0.8342 (3)	0.1127 (3)	0.0675 (2)	0.0203 (7)	
H4	0.9089	0.1160	0.0887	0.024*	
H4A	0.7797	0.0369	0.0506	0.024*	
N5	0.5071 (3)	0.7408 (3)	−0.1641 (2)	0.0194 (7)	
H5	0.4659	0.7140	−0.2303	0.023*	
H5A	0.5420	0.8239	−0.1330	0.023*	
N6	0.7913 (3)	0.8925 (3)	0.2088 (2)	0.0236 (7)	
H6	0.8574	0.8985	0.1874	0.028*	
H6A	0.7870	0.9660	0.2301	0.028*	
N7	0.4558 (3)	0.9259 (3)	0.3788 (2)	0.0208 (7)	
H7	0.4972	0.9475	0.4436	0.025*	
H7A	0.4733	0.9957	0.3737	0.025*	
N8	0.1709 (3)	0.7980 (3)	0.0124 (2)	0.0210 (7)	
H8	0.1111	0.8127	0.0370	0.025*	
H8A	0.2232	0.8698	0.0195	0.025*	
C1	0.3559 (4)	0.2285 (4)	−0.0483 (3)	0.0234 (8)	
C2	0.2972 (4)	0.0906 (4)	−0.0983 (3)	0.0209 (8)	
H2B	0.2414	0.0600	−0.1668	0.025*	
C3	0.2244 (4)	0.0447 (4)	−0.0382 (3)	0.0229 (8)	
C4	0.4041 (5)	0.2698 (4)	−0.1216 (3)	0.0317 (10)	
H4C	0.3372	0.2462	−0.1794	0.047*	
H4D	0.4570	0.2316	−0.1458	0.047*	
H4E	0.4487	0.3578	−0.0863	0.047*	
C5	0.2723 (4)	0.2883 (4)	−0.0111 (4)	0.0348 (10)	
H5C	0.3125	0.3756	0.0137	0.052*	
H5D	0.2521	0.2734	0.0439	0.052*	
H5E	0.1992	0.2540	−0.0672	0.052*	
C6	0.4359 (4)	0.3684 (4)	0.3796 (3)	0.0265 (9)	
C7	0.3745 (4)	0.2393 (4)	0.3644 (3)	0.0218 (8)	
H7B	0.3023	0.2338	0.3869	0.026*	
C8	0.4521 (4)	0.2079 (4)	0.4268 (3)	0.0258 (9)	
C9	0.3439 (5)	0.4063 (5)	0.3404 (4)	0.0391 (12)	
H9C	0.2943	0.4180	0.3849	0.059*	
H9D	0.2932	0.3432	0.2716	0.059*	
H9E	0.3849	0.4821	0.3394	0.059*	
C10	0.5084 (5)	0.4600 (4)	0.4895 (4)	0.0350 (11)	
H10C	0.5444	0.5407	0.4955	0.052*	
H10D	0.5716	0.4383	0.5120	0.052*	
H10E	0.4561	0.4600	0.5319	0.052*	
C11	0.9312 (4)	0.4296 (4)	0.4314 (3)	0.0254 (9)	
C12	0.9076 (4)	0.3144 (4)	0.4416 (3)	0.0249 (9)	
H12B	0.9589	0.3384	0.5101	0.030*	
C13	0.9386 (4)	0.2236 (4)	0.3631 (3)	0.0213 (8)	
C14	0.9306 (5)	0.5310 (5)	0.5255 (4)	0.0455 (13)	
H14C	1.0004	0.5587	0.5825	0.068*	
H14D	0.8576	0.5010	0.5419	0.068*	
H14E	0.9334	0.5983	0.5126	0.068*	
C15	1.0490 (4)	0.4763 (4)	0.4098 (4)	0.0375 (11)	
H15C	1.0545	0.5440	0.3965	0.056*	
H15D	1.0513	0.4111	0.3505	0.056*	
H15E	1.1163	0.5036	0.4687	0.056*	
C16	0.8366 (3)	0.2537 (3)	−0.0042 (3)	0.0202 (8)	
C17	0.8233 (3)	0.1252 (4)	−0.0265 (3)	0.0207 (8)	
H17B	0.8911	0.1137	−0.0508	0.025*	
C18	0.7080 (3)	0.0242 (3)	−0.1089 (3)	0.0200 (8)	
C19	0.9684 (4)	0.3435 (4)	0.0515 (3)	0.0288 (9)	
H19C	1.0158	0.3253	0.0060	0.043*	
H19D	0.9979	0.3364	0.1113	0.043*	
H19E	0.9753	0.4261	0.0726	0.043*	
C20	0.7879 (4)	0.2604 (4)	−0.0998 (3)	0.0266 (9)	
H20C	0.7841	0.3388	−0.0803	0.040*	
H20D	0.7078	0.1955	−0.1387	0.040*	
H20E	0.8408	0.2513	−0.1415	0.040*	
C21	0.5636 (4)	0.5795 (3)	−0.1569 (3)	0.0206 (8)	
C22	0.6039 (3)	0.6977 (4)	−0.1639 (3)	0.0197 (8)	
H22B	0.6204	0.6801	−0.2298	0.024*	
C23	0.7192 (4)	0.7945 (4)	−0.0790 (3)	0.0220 (8)	
C24	0.4685 (4)	0.4759 (4)	−0.2548 (3)	0.0304 (10)	
H24C	0.5037	0.4590	−0.3099	0.046*	
H24D	0.4034	0.4984	−0.2712	0.046*	
H24E	0.4372	0.4037	−0.2464	0.046*	
C25	0.6677 (4)	0.5470 (4)	−0.1301 (4)	0.0303 (10)	
H25C	0.6361	0.4668	−0.1345	0.045*	
H25D	0.7201	0.6070	−0.0610	0.045*	
H25E	0.7132	0.5466	−0.1776	0.045*	
C26	0.7410 (4)	0.7371 (4)	0.2672 (3)	0.0253 (9)	
C27	0.8110 (4)	0.8710 (4)	0.2986 (3)	0.0215 (8)	
H27B	0.8978	0.8908	0.3235	0.026*	
C28	0.7823 (4)	0.9590 (4)	0.3831 (3)	0.0262 (9)	
C29	0.7994 (4)	0.6606 (5)	0.2034 (4)	0.0359 (11)	
H29C	0.8081	0.6779	0.1471	0.054*	
H29D	0.7489	0.5746	0.1766	0.054*	
H29E	0.8781	0.6808	0.2461	0.054*	
C30	0.7311 (5)	0.7148 (5)	0.3579 (4)	0.0364 (11)	
H30C	0.8112	0.7447	0.4037	0.055*	
H30D	0.6888	0.6279	0.3339	0.055*	
H30E	0.6870	0.7573	0.3941	0.055*	
C31	0.2743 (4)	0.7331 (4)	0.3133 (3)	0.0211 (8)	
C32	0.3264 (4)	0.8728 (4)	0.3654 (3)	0.0190 (8)	
H32B	0.3172	0.9012	0.4345	0.023*	
C33	0.2592 (4)	0.9188 (3)	0.3094 (3)	0.0210 (8)	
C34	0.3183 (4)	0.6923 (4)	0.3870 (3)	0.0283 (9)	
H34C	0.2912	0.7207	0.4461	0.042*	
H34D	0.4057	0.7261	0.4088	0.042*	
H34E	0.2861	0.6038	0.3532	0.042*	
C35	0.1378 (4)	0.6754 (4)	0.2741 (3)	0.0266 (9)	
H35C	0.1088	0.5872	0.2401	0.040*	
H35D	0.1105	0.7010	0.2260	0.040*	
H35E	0.1067	0.7011	0.3311	0.040*	
C36	0.1010 (3)	0.5834 (3)	−0.1193 (3)	0.0211 (8)	
C37	0.1163 (3)	0.7115 (3)	−0.0981 (3)	0.0174 (7)	
H37B	0.0347	0.7079	−0.1193	0.021*	
C38	0.1877 (3)	0.7582 (3)	−0.1584 (3)	0.0176 (7)	
C39	−0.0021 (4)	0.5281 (4)	−0.0858 (4)	0.0327 (10)	
H39C	−0.0772	0.5156	−0.1285	0.049*	
H39D	0.0108	0.5827	−0.0150	0.049*	
H39E	−0.0059	0.4503	−0.0926	0.049*	
C40	0.0796 (4)	0.4998 (4)	−0.2302 (3)	0.0306 (10)	
H40C	0.0787	0.4226	−0.2379	0.046*	
H40D	0.1437	0.5373	−0.2526	0.046*	
H40E	0.0027	0.4857	−0.2714	0.046*	
O17	0.2360 (3)	0.7792 (3)	0.6258 (3)	0.0368 (8)	
H17F	0.295 (4)	0.846 (3)	0.647 (3)	0.044*	
H17G	0.213 (4)	0.779 (4)	0.676 (3)	0.044*	
O18	0.3581 (3)	0.6315 (3)	0.6191 (3)	0.0376 (8)	
H18F	0.323 (4)	0.677 (4)	0.632 (4)	0.045*	
H18G	0.422 (3)	0.665 (4)	0.609 (4)	0.045*	
O19	0.9512 (3)	0.0057 (3)	0.7877 (3)	0.0372 (8)	
H19F	0.881 (2)	−0.021 (5)	0.792 (4)	0.045*	
H19G	0.997 (3)	−0.010 (5)	0.823 (4)	0.045*	
O20	0.5778 (3)	0.7806 (3)	0.6202 (2)	0.0372 (8)	
H20F	0.635 (3)	0.768 (5)	0.601 (3)	0.045*	
H20G	0.603 (4)	0.826 (4)	0.6846 (16)	0.045*	
O21	0.7559 (3)	0.7161 (4)	0.5734 (3)	0.0460 (9)	
H21F	0.782 (4)	0.694 (5)	0.615 (4)	0.055*	
H21G	0.818 (3)	0.772 (4)	0.576 (4)	0.055*	
O22	0.8781 (3)	0.6974 (4)	0.7329 (3)	0.0414 (9)	
H22F	0.859 (4)	0.728 (5)	0.786 (3)	0.050*	
H22G	0.947 (3)	0.749 (4)	0.742 (4)	0.050*	
O23	0.5672 (3)	0.9673 (3)	0.5820 (3)	0.0363 (8)	
H23F	0.563 (4)	0.904 (3)	0.585 (4)	0.044*	
H23G	0.641 (2)	1.013 (4)	0.600 (4)	0.044*	
O24	0.0002 (3)	0.2202 (4)	0.7741 (3)	0.0449 (9)	
H24F	−0.012 (5)	0.157 (4)	0.779 (5)	0.054*	
H24G	0.060 (4)	0.235 (5)	0.753 (4)	0.054*	
O25	0.7894 (3)	0.1475 (3)	0.6288 (3)	0.0416 (8)	
H25F	0.858 (3)	0.178 (5)	0.674 (3)	0.050*	
H25G	0.802 (4)	0.116 (5)	0.572 (2)	0.050*	
O26	0.4061 (3)	0.0111 (3)	0.6861 (3)	0.0386 (8)	
H26F	0.464 (3)	0.007 (5)	0.663 (4)	0.046*	
H26G	0.362 (4)	0.026 (5)	0.649 (4)	0.046*	
O27	0.0682 (3)	0.0366 (3)	0.2512 (2)	0.0374 (8)	
H27F	0.040 (4)	0.077 (4)	0.293 (3)	0.045*	
H27G	0.115 (4)	0.018 (5)	0.282 (3)	0.045*	
O28	0.9639 (3)	0.8198 (4)	0.0690 (3)	0.0405 (8)	
H28F	0.930 (5)	0.835 (5)	0.028 (3)	0.049*	
H28G	0.996 (5)	0.875 (4)	0.1274 (18)	0.049*	
O29	0.0768 (3)	0.1372 (4)	0.1199 (3)	0.0439 (9)	
H29F	0.078 (6)	0.096 (4)	0.059 (2)	0.053*	
H29G	0.069 (6)	0.091 (4)	0.147 (4)	0.053*	
O30	0.9422 (4)	0.9185 (4)	0.5849 (3)	0.0563 (11)	
H30F	0.961 (6)	0.983 (3)	0.578 (4)	0.068*	
H30G	0.954 (6)	0.940 (5)	0.6485 (19)	0.068*	
O31	0.2735 (4)	0.0901 (4)	0.5859 (3)	0.0523 (10)	
H31F	0.213 (4)	0.025 (4)	0.549 (4)	0.063*	
H31G	0.315 (5)	0.109 (5)	0.550 (4)	0.063*	
O32	0.1683 (6)	0.2489 (5)	0.6778 (5)	0.094 (2)	
H32F	0.144 (4)	0.263 (5)	0.626 (4)	0.113*	
H32G	0.202 (8)	0.202 (7)	0.651 (6)	0.113*	
O33	0.0993 (5)	0.8375 (5)	0.4988 (4)	0.0651 (13)	
H33F	0.111 (6)	0.797 (6)	0.530 (5)	0.078*	
H33G	0.056 (6)	0.871 (6)	0.529 (5)	0.078*	
O34	0.2066 (5)	0.3892 (5)	0.5768 (4)	0.0731 (15)	
H34F	0.267 (4)	0.450 (5)	0.587 (5)	0.088*	
H34G	0.164 (5)	0.353 (6)	0.514 (2)	0.088*	
O35A	0.6203 (8)	0.2636 (7)	0.6738 (6)	0.0473 (18)	0.50
H35F	0.673 (8)	0.236 (11)	0.662 (8)	0.057*	0.50
H35G	0.562 (7)	0.227 (10)	0.620 (5)	0.057*	0.50
O35B	0.7105 (7)	0.3189 (8)	0.6173 (6)	0.0428 (18)	0.50
H35H	0.645 (5)	0.323 (10)	0.613 (9)	0.051*	0.50
H35I	0.710 (9)	0.258 (7)	0.622 (10)	0.051*	0.50
O36A	0.7793 (9)	0.4317 (7)	0.6403 (6)	0.060 (3)	0.50
H36F	0.790 (12)	0.506 (4)	0.669 (10)	0.072*	0.50
H36G	0.736 (12)	0.395 (9)	0.666 (10)	0.072*	0.50

### Atomic displacement parameters (Å^2^)

**Table:**

	*U*^11^	*U*^22^	*U*^33^	*U*^12^	*U*^13^	*U*^23^
Pd1	0.01898 (15)	0.01577 (14)	0.01867 (14)	0.00661 (11)	0.00782 (11)	0.00872 (11)
Pd2	0.01978 (15)	0.01643 (14)	0.01862 (14)	0.00832 (11)	0.00777 (11)	0.00939 (11)
Pd3	0.02208 (15)	0.02032 (15)	0.02266 (15)	0.01175 (12)	0.01158 (12)	0.01195 (12)
Pd4	0.01941 (15)	0.01653 (14)	0.01521 (13)	0.00718 (11)	0.00666 (10)	0.00626 (11)
Pd5	0.01712 (14)	0.01635 (13)	0.01776 (13)	0.00712 (11)	0.00635 (10)	0.00918 (11)
Pd6	0.01805 (14)	0.01675 (13)	0.01560 (13)	0.00801 (11)	0.00604 (10)	0.00728 (11)
Pd7	0.02024 (15)	0.01885 (14)	0.01730 (14)	0.01009 (11)	0.00649 (11)	0.00875 (12)
Pd8	0.02006 (14)	0.01697 (14)	0.01576 (13)	0.00908 (11)	0.00712 (10)	0.00920 (11)
S1	0.0235 (5)	0.0168 (4)	0.0221 (5)	0.0086 (4)	0.0102 (4)	0.0104 (4)
S2	0.0233 (5)	0.0166 (4)	0.0219 (4)	0.0090 (4)	0.0090 (4)	0.0098 (4)
S3	0.0230 (5)	0.0175 (4)	0.0194 (4)	0.0084 (4)	0.0091 (4)	0.0074 (4)
S4	0.0221 (5)	0.0164 (4)	0.0191 (4)	0.0065 (4)	0.0075 (3)	0.0078 (4)
S5	0.0205 (5)	0.0167 (4)	0.0191 (4)	0.0081 (3)	0.0059 (3)	0.0082 (4)
S6	0.0226 (5)	0.0199 (4)	0.0191 (4)	0.0102 (4)	0.0081 (3)	0.0103 (4)
S7	0.0225 (5)	0.0186 (4)	0.0198 (4)	0.0110 (4)	0.0076 (3)	0.0095 (4)
S8	0.0210 (5)	0.0170 (4)	0.0194 (4)	0.0082 (4)	0.0083 (3)	0.0104 (4)
O1	0.0247 (15)	0.0210 (14)	0.0206 (14)	0.0049 (11)	0.0053 (11)	0.0114 (12)
O2	0.0210 (15)	0.050 (2)	0.0358 (17)	0.0146 (14)	0.0108 (12)	0.0262 (16)
O3	0.0333 (17)	0.0367 (17)	0.057 (2)	0.0233 (14)	0.0292 (15)	0.0353 (16)
O4	0.057 (2)	0.083 (3)	0.037 (2)	0.045 (2)	0.0266 (17)	0.041 (2)
O5	0.0362 (17)	0.0256 (15)	0.0163 (13)	0.0162 (13)	0.0044 (11)	0.0057 (12)
O6	0.042 (2)	0.056 (2)	0.0342 (19)	0.0325 (18)	−0.0008 (15)	−0.0023 (17)
O7	0.0176 (13)	0.0153 (13)	0.0292 (15)	0.0049 (10)	0.0078 (11)	0.0054 (11)
O8	0.0266 (15)	0.0299 (16)	0.0197 (14)	0.0110 (13)	0.0062 (11)	0.0004 (12)
O9	0.0273 (15)	0.0221 (14)	0.0219 (14)	0.0072 (11)	0.0127 (11)	0.0111 (12)
O10	0.0231 (16)	0.0492 (19)	0.0283 (16)	0.0150 (14)	0.0128 (12)	0.0174 (15)
O11	0.0262 (15)	0.0218 (14)	0.0311 (16)	0.0112 (12)	0.0068 (12)	0.0075 (13)
O12	0.040 (2)	0.072 (3)	0.0229 (17)	0.0284 (19)	−0.0025 (14)	−0.0044 (18)
O13	0.0420 (18)	0.0267 (15)	0.0194 (14)	0.0219 (14)	0.0109 (12)	0.0138 (12)
O14	0.062 (2)	0.064 (2)	0.0338 (18)	0.055 (2)	0.0265 (16)	0.0299 (17)
O15	0.0225 (14)	0.0262 (15)	0.0283 (15)	0.0109 (12)	0.0103 (11)	0.0191 (12)
O16	0.0275 (16)	0.0426 (18)	0.0295 (15)	0.0192 (13)	0.0126 (12)	0.0278 (14)
N1	0.0219 (17)	0.0180 (16)	0.0216 (16)	0.0065 (13)	0.0098 (13)	0.0104 (13)
N2	0.0240 (18)	0.0231 (17)	0.0253 (17)	0.0060 (14)	0.0113 (14)	0.0135 (15)
N3	0.038 (2)	0.043 (2)	0.034 (2)	0.0275 (18)	0.0237 (16)	0.0291 (18)
N4	0.0210 (16)	0.0196 (16)	0.0208 (16)	0.0101 (13)	0.0092 (13)	0.0080 (13)
N5	0.0165 (15)	0.0218 (16)	0.0188 (15)	0.0073 (13)	0.0074 (12)	0.0087 (13)
N6	0.0226 (17)	0.0241 (17)	0.0196 (16)	0.0041 (13)	0.0060 (13)	0.0109 (14)
N7	0.0251 (17)	0.0187 (16)	0.0194 (16)	0.0102 (13)	0.0054 (13)	0.0096 (13)
N8	0.0234 (17)	0.0212 (16)	0.0224 (16)	0.0105 (13)	0.0120 (13)	0.0113 (14)
C1	0.029 (2)	0.022 (2)	0.024 (2)	0.0120 (16)	0.0071 (16)	0.0146 (17)
C2	0.025 (2)	0.027 (2)	0.0171 (18)	0.0140 (16)	0.0090 (15)	0.0131 (16)
C3	0.023 (2)	0.022 (2)	0.0225 (19)	0.0092 (16)	0.0086 (15)	0.0095 (16)
C4	0.051 (3)	0.023 (2)	0.029 (2)	0.015 (2)	0.0132 (19)	0.0197 (18)
C5	0.039 (3)	0.033 (2)	0.042 (3)	0.024 (2)	0.014 (2)	0.020 (2)
C6	0.031 (2)	0.023 (2)	0.029 (2)	0.0149 (18)	0.0151 (17)	0.0095 (17)
C7	0.0203 (19)	0.023 (2)	0.0229 (19)	0.0114 (16)	0.0115 (15)	0.0083 (16)
C8	0.025 (2)	0.021 (2)	0.031 (2)	0.0094 (16)	0.0098 (17)	0.0129 (17)
C9	0.041 (3)	0.044 (3)	0.058 (3)	0.033 (2)	0.030 (2)	0.032 (3)
C10	0.047 (3)	0.027 (2)	0.030 (2)	0.015 (2)	0.021 (2)	0.0093 (19)
C11	0.025 (2)	0.022 (2)	0.0207 (19)	0.0097 (16)	0.0010 (15)	0.0043 (16)
C12	0.024 (2)	0.033 (2)	0.0173 (19)	0.0167 (18)	0.0050 (15)	0.0083 (17)
C13	0.0222 (19)	0.0197 (19)	0.0174 (18)	0.0094 (15)	0.0030 (14)	0.0053 (15)
C14	0.064 (4)	0.033 (3)	0.024 (2)	0.028 (3)	0.002 (2)	−0.002 (2)
C15	0.024 (2)	0.036 (3)	0.043 (3)	0.0050 (19)	0.0066 (19)	0.017 (2)
C16	0.0216 (19)	0.0176 (18)	0.0190 (18)	0.0039 (15)	0.0084 (14)	0.0091 (15)
C17	0.0177 (19)	0.0222 (19)	0.0198 (19)	0.0048 (15)	0.0080 (14)	0.0099 (16)
C18	0.0191 (19)	0.0199 (18)	0.0218 (19)	0.0088 (15)	0.0060 (14)	0.0102 (16)
C19	0.021 (2)	0.027 (2)	0.028 (2)	0.0012 (16)	0.0053 (16)	0.0099 (18)
C20	0.030 (2)	0.027 (2)	0.023 (2)	0.0080 (18)	0.0112 (17)	0.0152 (18)
C21	0.024 (2)	0.0187 (18)	0.0196 (18)	0.0116 (16)	0.0088 (15)	0.0073 (15)
C22	0.0201 (19)	0.0228 (19)	0.0169 (18)	0.0093 (15)	0.0081 (14)	0.0090 (16)
C23	0.027 (2)	0.0194 (19)	0.0212 (19)	0.0105 (16)	0.0061 (15)	0.0103 (16)
C24	0.040 (3)	0.020 (2)	0.022 (2)	0.0063 (18)	0.0077 (18)	0.0076 (17)
C25	0.035 (2)	0.036 (2)	0.036 (2)	0.026 (2)	0.0180 (19)	0.021 (2)
C26	0.026 (2)	0.027 (2)	0.025 (2)	0.0132 (17)	0.0029 (16)	0.0146 (18)
C27	0.0210 (19)	0.0220 (19)	0.0197 (18)	0.0094 (16)	0.0053 (14)	0.0083 (16)
C28	0.027 (2)	0.024 (2)	0.023 (2)	0.0088 (17)	0.0052 (16)	0.0087 (17)
C29	0.032 (2)	0.038 (3)	0.045 (3)	0.025 (2)	0.010 (2)	0.020 (2)
C30	0.042 (3)	0.037 (3)	0.037 (3)	0.016 (2)	0.007 (2)	0.026 (2)
C31	0.026 (2)	0.0209 (19)	0.0189 (18)	0.0138 (16)	0.0083 (15)	0.0080 (15)
C32	0.025 (2)	0.0220 (19)	0.0166 (17)	0.0142 (16)	0.0104 (14)	0.0110 (15)
C33	0.026 (2)	0.0161 (18)	0.0185 (18)	0.0102 (15)	0.0037 (15)	0.0063 (15)
C34	0.036 (2)	0.031 (2)	0.030 (2)	0.0193 (19)	0.0162 (18)	0.0196 (19)
C35	0.028 (2)	0.030 (2)	0.031 (2)	0.0139 (18)	0.0149 (17)	0.0183 (19)
C36	0.022 (2)	0.0158 (18)	0.0212 (19)	0.0032 (15)	0.0026 (15)	0.0094 (16)
C37	0.0155 (18)	0.0196 (18)	0.0205 (18)	0.0055 (14)	0.0063 (14)	0.0139 (16)
C38	0.0199 (19)	0.0171 (17)	0.0180 (17)	0.0086 (14)	0.0080 (14)	0.0088 (15)
C39	0.025 (2)	0.032 (2)	0.036 (2)	0.0030 (18)	0.0062 (18)	0.020 (2)
C40	0.037 (3)	0.024 (2)	0.020 (2)	0.0036 (18)	0.0021 (17)	0.0098 (18)
O17	0.040 (2)	0.0383 (19)	0.0361 (18)	0.0147 (15)	0.0203 (15)	0.0197 (16)
O18	0.040 (2)	0.0367 (19)	0.0373 (19)	0.0171 (15)	0.0110 (15)	0.0177 (16)
O19	0.0335 (18)	0.051 (2)	0.0362 (19)	0.0196 (16)	0.0129 (15)	0.0269 (17)
O20	0.0357 (19)	0.0325 (18)	0.0287 (17)	0.0126 (15)	0.0022 (13)	0.0051 (14)
O21	0.047 (2)	0.057 (2)	0.040 (2)	0.0277 (18)	0.0133 (17)	0.0246 (19)
O22	0.0326 (18)	0.060 (2)	0.0297 (17)	0.0237 (17)	0.0130 (14)	0.0149 (17)
O23	0.0358 (18)	0.044 (2)	0.0336 (17)	0.0187 (15)	0.0064 (14)	0.0221 (16)
O24	0.037 (2)	0.048 (2)	0.062 (3)	0.0216 (18)	0.0201 (18)	0.032 (2)
O25	0.047 (2)	0.043 (2)	0.0327 (18)	0.0220 (17)	0.0151 (15)	0.0127 (17)
O26	0.043 (2)	0.050 (2)	0.039 (2)	0.0259 (18)	0.0221 (16)	0.0283 (18)
O27	0.050 (2)	0.051 (2)	0.0245 (15)	0.0407 (17)	0.0119 (14)	0.0145 (15)
O28	0.0282 (18)	0.056 (2)	0.0349 (19)	0.0183 (16)	0.0068 (14)	0.0201 (18)
O29	0.0353 (19)	0.059 (2)	0.047 (2)	0.0236 (18)	0.0222 (17)	0.0268 (19)
O30	0.061 (3)	0.067 (3)	0.041 (2)	0.033 (2)	0.0171 (19)	0.020 (2)
O31	0.071 (3)	0.071 (3)	0.043 (2)	0.046 (2)	0.032 (2)	0.036 (2)
O32	0.113 (5)	0.093 (4)	0.161 (6)	0.074 (4)	0.103 (4)	0.093 (4)
O33	0.072 (3)	0.087 (4)	0.055 (3)	0.046 (3)	0.028 (2)	0.038 (3)
O34	0.068 (3)	0.063 (3)	0.055 (3)	−0.001 (2)	−0.010 (2)	0.030 (3)
O35A	0.053 (5)	0.046 (4)	0.045 (4)	0.025 (4)	0.012 (3)	0.022 (4)
O35B	0.055 (5)	0.051 (5)	0.031 (4)	0.026 (4)	0.017 (3)	0.023 (3)
O36A	0.096 (7)	0.037 (4)	0.045 (5)	0.017 (5)	0.049 (5)	0.017 (4)

### Geometric parameters (Å, º)

**Table:**

Pd1—O7	2.079 (3)	C12—C13	1.518 (5)
Pd1—N1	2.089 (3)	C12—H12B	1.0000
Pd1—S1	2.2315 (10)	C14—H14C	0.9800
Pd1—S4	2.2867 (10)	C14—H14D	0.9800
Pd2—O1	2.086 (3)	C14—H14E	0.9800
Pd2—N2	2.087 (3)	C15—H15C	0.9800
Pd2—S2	2.2261 (11)	C15—H15D	0.9800
Pd2—S1	2.2871 (10)	C15—H15E	0.9800
Pd3—N3	2.070 (4)	C16—C20	1.536 (6)
Pd3—O3	2.085 (3)	C16—C19	1.540 (5)
Pd3—S3	2.2302 (10)	C16—C17	1.547 (6)
Pd3—S2	2.2843 (11)	C17—C18	1.527 (5)
Pd4—N4	2.080 (3)	C17—H17B	1.0000
Pd4—O5	2.088 (3)	C19—H19C	0.9800
Pd4—S4	2.2338 (11)	C19—H19D	0.9800
Pd4—S3	2.2876 (10)	C19—H19E	0.9800
Pd5—O15	2.082 (3)	C20—H20C	0.9800
Pd5—N5	2.097 (3)	C20—H20D	0.9800
Pd5—S5	2.2285 (11)	C20—H20E	0.9800
Pd5—S8	2.2869 (9)	C21—C24	1.522 (5)
Pd6—N6	2.089 (3)	C21—C22	1.539 (6)
Pd6—O9	2.095 (3)	C21—C25	1.541 (6)
Pd6—S6	2.2306 (10)	C22—C23	1.527 (5)
Pd6—S5	2.2819 (10)	C22—H22B	1.0000
Pd7—N7	2.076 (3)	C24—H24C	0.9800
Pd7—O11	2.080 (3)	C24—H24D	0.9800
Pd7—S7	2.2427 (10)	C24—H24E	0.9800
Pd7—S6	2.2894 (10)	C25—H25C	0.9800
Pd8—N8	2.065 (3)	C25—H25D	0.9800
Pd8—O13	2.068 (3)	C25—H25E	0.9800
Pd8—S8	2.2382 (10)	C26—C30	1.532 (6)
Pd8—S7	2.2968 (10)	C26—C27	1.536 (6)
S1—C1	1.861 (4)	C26—C29	1.544 (6)
S2—C6	1.863 (4)	C27—C28	1.529 (6)
S3—C11	1.863 (4)	C27—H27B	1.0000
S4—C16	1.856 (4)	C29—H29C	0.9800
S5—C21	1.864 (4)	C29—H29D	0.9800
S6—C26	1.860 (4)	C29—H29E	0.9800
S7—C31	1.865 (4)	C30—H30C	0.9800
S8—C36	1.864 (4)	C30—H30D	0.9800
O1—C3	1.282 (5)	C30—H30E	0.9800
O2—C3	1.226 (5)	C31—C34	1.528 (6)
O3—C8	1.291 (5)	C31—C35	1.529 (6)
O4—C8	1.214 (5)	C31—C32	1.548 (5)
O5—C13	1.285 (5)	C32—C33	1.525 (6)
O6—C13	1.221 (5)	C32—H32B	1.0000
O7—C18	1.273 (5)	C34—H34C	0.9800
O8—C18	1.228 (5)	C34—H34D	0.9800
O9—C23	1.286 (5)	C34—H34E	0.9800
O10—C23	1.233 (5)	C35—H35C	0.9800
O11—C28	1.263 (5)	C35—H35D	0.9800
O12—C28	1.225 (5)	C35—H35E	0.9800
O13—C33	1.269 (5)	C36—C40	1.524 (6)
O14—C33	1.238 (5)	C36—C39	1.527 (6)
O15—C38	1.280 (5)	C36—C37	1.538 (5)
O16—C38	1.238 (5)	C37—C38	1.533 (5)
N1—C2	1.502 (5)	C37—H37B	1.0000
N1—H1	0.9200	C39—H39C	0.9800
N1—H1A	0.9200	C39—H39D	0.9800
N2—C7	1.494 (5)	C39—H39E	0.9800
N2—H2	0.9200	C40—H40C	0.9800
N2—H2A	0.9200	C40—H40D	0.9800
N3—C12	1.501 (6)	C40—H40E	0.9800
N3—H3	0.9200	O17—H17F	0.847 (19)
N3—H3A	0.9200	O17—H17G	0.856 (19)
N4—C17	1.491 (5)	O18—H18F	0.841 (19)
N4—H4	0.9200	O18—H18G	0.852 (19)
N4—H4A	0.9200	O19—H19F	0.851 (19)
N5—C22	1.490 (5)	O19—H19G	0.854 (19)
N5—H5	0.9200	O20—H20F	0.845 (19)
N5—H5A	0.9200	O20—H20G	0.858 (19)
N6—C27	1.501 (5)	O21—H21F	0.863 (19)
N6—H6	0.9200	O21—H21G	0.869 (19)
N6—H6A	0.9200	O22—H22F	0.854 (19)
N7—C32	1.484 (5)	O22—H22G	0.859 (19)
N7—H7	0.9200	O23—H23F	0.851 (19)
N7—H7A	0.9200	O23—H23G	0.854 (19)
N8—C37	1.491 (5)	O24—H24F	0.849 (19)
N8—H8	0.9200	O24—H24G	0.851 (19)
N8—H8A	0.9200	O25—H25F	0.865 (19)
C1—C2	1.529 (6)	O25—H25G	0.852 (19)
C1—C4	1.539 (6)	O26—H26F	0.860 (19)
C1—C5	1.541 (6)	O26—H26G	0.851 (19)
C2—C3	1.535 (5)	O27—H27F	0.847 (19)
C2—H2B	1.0000	O27—H27G	0.844 (19)
C4—H4C	0.9800	O28—H28F	0.822 (19)
C4—H4D	0.9800	O28—H28G	0.821 (19)
C4—H4E	0.9800	O29—H29F	0.868 (19)
C5—H5C	0.9800	O29—H29G	0.854 (19)
C5—H5D	0.9800	O30—H30F	0.861 (19)
C5—H5E	0.9800	O30—H30G	0.864 (19)
C6—C10	1.521 (6)	O31—H31F	0.839 (19)
C6—C9	1.525 (7)	O31—H31G	0.858 (19)
C6—C7	1.540 (6)	O32—H32F	0.917 (19)
C7—C8	1.518 (6)	O32—H32G	0.86 (2)
C7—H7B	1.0000	O33—H33F	0.875 (19)
C9—H9C	0.9800	O33—H33G	0.87 (2)
C9—H9D	0.9800	O34—H34F	0.85 (2)
C9—H9E	0.9800	O34—H34G	0.86 (2)
C10—H10C	0.9800	O35A—H35F	0.85 (2)
C10—H10D	0.9800	O35A—H35G	0.85 (2)
C10—H10E	0.9800	O35B—H35H	0.83 (2)
C11—C14	1.524 (6)	O35B—H35I	0.84 (2)
C11—C12	1.537 (6)	O36A—H36F	0.85 (2)
C11—C15	1.539 (6)	O36A—H36G	0.85 (2)
			
O7—Pd1—N1	85.63 (12)	C14—C11—C15	108.8 (4)
O7—Pd1—S1	171.94 (8)	C12—C11—C15	113.5 (4)
N1—Pd1—S1	86.31 (9)	C14—C11—S3	106.2 (3)
O7—Pd1—S4	98.20 (8)	C12—C11—S3	109.0 (3)
N1—Pd1—S4	168.53 (10)	C15—C11—S3	107.6 (3)
S1—Pd1—S4	89.76 (4)	N3—C12—C13	110.1 (3)
O1—Pd2—N2	86.93 (12)	N3—C12—C11	110.2 (3)
O1—Pd2—S2	171.92 (8)	C13—C12—C11	112.9 (4)
N2—Pd2—S2	85.00 (10)	N3—C12—H12B	107.8
O1—Pd2—S1	96.38 (8)	C13—C12—H12B	107.8
N2—Pd2—S1	171.42 (11)	C11—C12—H12B	107.8
S2—Pd2—S1	91.67 (4)	O6—C13—O5	122.2 (4)
N3—Pd3—O3	85.09 (14)	O6—C13—C12	118.4 (4)
N3—Pd3—S3	86.53 (10)	O5—C13—C12	119.4 (4)
O3—Pd3—S3	170.89 (9)	C11—C14—H14C	109.5
N3—Pd3—S2	167.45 (12)	C11—C14—H14D	109.5
O3—Pd3—S2	98.63 (9)	H14C—C14—H14D	109.5
S3—Pd3—S2	90.31 (4)	C11—C14—H14E	109.5
N4—Pd4—O5	85.91 (12)	H14C—C14—H14E	109.5
N4—Pd4—S4	85.40 (10)	H14D—C14—H14E	109.5
O5—Pd4—S4	171.31 (8)	C11—C15—H15C	109.5
N4—Pd4—S3	170.89 (9)	C11—C15—H15D	109.5
O5—Pd4—S3	97.34 (8)	H15C—C15—H15D	109.5
S4—Pd4—S3	91.28 (4)	C11—C15—H15E	109.5
O15—Pd5—N5	86.31 (12)	H15C—C15—H15E	109.5
O15—Pd5—S5	172.13 (8)	H15D—C15—H15E	109.5
N5—Pd5—S5	85.87 (10)	C20—C16—C19	111.0 (3)
O15—Pd5—S8	97.94 (8)	C20—C16—C17	112.9 (3)
N5—Pd5—S8	169.40 (9)	C19—C16—C17	109.6 (4)
S5—Pd5—S8	89.92 (4)	C20—C16—S4	107.7 (3)
N6—Pd6—O9	86.45 (12)	C19—C16—S4	107.0 (3)
N6—Pd6—S6	85.64 (10)	C17—C16—S4	108.5 (3)
O9—Pd6—S6	172.08 (8)	N4—C17—C18	109.7 (3)
N6—Pd6—S5	169.28 (11)	N4—C17—C16	111.1 (3)
O9—Pd6—S5	96.66 (8)	C18—C17—C16	114.0 (4)
S6—Pd6—S5	91.20 (4)	N4—C17—H17B	107.2
N7—Pd7—O11	84.28 (12)	C18—C17—H17B	107.2
N7—Pd7—S7	86.56 (9)	C16—C17—H17B	107.2
O11—Pd7—S7	170.76 (9)	O8—C18—O7	123.4 (4)
N7—Pd7—S6	170.75 (10)	O8—C18—C17	117.0 (4)
O11—Pd7—S6	98.17 (9)	O7—C18—C17	119.6 (3)
S7—Pd7—S6	91.07 (4)	C16—C19—H19C	109.5
N8—Pd8—O13	84.85 (12)	C16—C19—H19D	109.5
N8—Pd8—S8	85.18 (9)	H19C—C19—H19D	109.5
O13—Pd8—S8	170.03 (9)	C16—C19—H19E	109.5
N8—Pd8—S7	170.92 (9)	H19C—C19—H19E	109.5
O13—Pd8—S7	96.17 (8)	H19D—C19—H19E	109.5
S8—Pd8—S7	93.65 (4)	C16—C20—H20C	109.5
C1—S1—Pd1	97.60 (14)	C16—C20—H20D	109.5
C1—S1—Pd2	104.04 (13)	H20C—C20—H20D	109.5
Pd1—S1—Pd2	97.42 (4)	C16—C20—H20E	109.5
C6—S2—Pd2	97.34 (14)	H20C—C20—H20E	109.5
C6—S2—Pd3	102.37 (15)	H20D—C20—H20E	109.5
Pd2—S2—Pd3	100.53 (4)	C24—C21—C22	110.5 (3)
C11—S3—Pd3	97.93 (14)	C24—C21—C25	110.5 (4)
C11—S3—Pd4	103.18 (14)	C22—C21—C25	113.1 (3)
Pd3—S3—Pd4	96.90 (4)	C24—C21—S5	106.9 (3)
C16—S4—Pd4	98.06 (14)	C22—C21—S5	108.0 (3)
C16—S4—Pd1	101.06 (12)	C25—C21—S5	107.6 (3)
Pd4—S4—Pd1	102.42 (4)	N5—C22—C23	111.5 (3)
C21—S5—Pd5	98.24 (13)	N5—C22—C21	110.6 (3)
C21—S5—Pd6	102.92 (12)	C23—C22—C21	111.3 (3)
Pd5—S5—Pd6	99.24 (4)	N5—C22—H22B	107.8
C26—S6—Pd6	97.13 (14)	C23—C22—H22B	107.8
C26—S6—Pd7	103.20 (14)	C21—C22—H22B	107.8
Pd6—S6—Pd7	99.03 (4)	O10—C23—O9	122.8 (4)
C31—S7—Pd7	97.98 (13)	O10—C23—C22	119.7 (4)
C31—S7—Pd8	102.32 (14)	O9—C23—C22	117.5 (4)
Pd7—S7—Pd8	99.57 (4)	C21—C24—H24C	109.5
C36—S8—Pd8	97.79 (13)	C21—C24—H24D	109.5
C36—S8—Pd5	102.08 (13)	H24C—C24—H24D	109.5
Pd8—S8—Pd5	101.17 (4)	C21—C24—H24E	109.5
C3—O1—Pd2	119.2 (3)	H24C—C24—H24E	109.5
C8—O3—Pd3	119.8 (3)	H24D—C24—H24E	109.5
C13—O5—Pd4	121.7 (3)	C21—C25—H25C	109.5
C18—O7—Pd1	127.7 (2)	C21—C25—H25D	109.5
C23—O9—Pd6	117.0 (2)	H25C—C25—H25D	109.5
C28—O11—Pd7	124.1 (3)	C21—C25—H25E	109.5
C33—O13—Pd8	127.9 (3)	H25C—C25—H25E	109.5
C38—O15—Pd5	127.3 (2)	H25D—C25—H25E	109.5
C2—N1—Pd1	116.6 (2)	C30—C26—C27	112.9 (4)
C2—N1—H1	108.1	C30—C26—C29	110.1 (4)
Pd1—N1—H1	108.1	C27—C26—C29	109.9 (4)
C2—N1—H1A	108.1	C30—C26—S6	108.5 (3)
Pd1—N1—H1A	108.1	C27—C26—S6	108.7 (3)
H1—N1—H1A	107.3	C29—C26—S6	106.6 (3)
C7—N2—Pd2	118.2 (2)	N6—C27—C28	108.8 (3)
C7—N2—H2	107.8	N6—C27—C26	110.5 (3)
Pd2—N2—H2	107.8	C28—C27—C26	114.7 (3)
C7—N2—H2A	107.8	N6—C27—H27B	107.5
Pd2—N2—H2A	107.8	C28—C27—H27B	107.5
H2—N2—H2A	107.1	C26—C27—H27B	107.5
C12—N3—Pd3	117.4 (3)	O12—C28—O11	123.1 (4)
C12—N3—H3	108.0	O12—C28—C27	118.1 (4)
Pd3—N3—H3	108.0	O11—C28—C27	118.7 (4)
C12—N3—H3A	108.0	C26—C29—H29C	109.5
Pd3—N3—H3A	108.0	C26—C29—H29D	109.5
H3—N3—H3A	107.2	H29C—C29—H29D	109.5
C17—N4—Pd4	118.0 (3)	C26—C29—H29E	109.5
C17—N4—H4	107.8	H29C—C29—H29E	109.5
Pd4—N4—H4	107.8	H29D—C29—H29E	109.5
C17—N4—H4A	107.8	C26—C30—H30C	109.5
Pd4—N4—H4A	107.8	C26—C30—H30D	109.5
H4—N4—H4A	107.1	H30C—C30—H30D	109.5
C22—N5—Pd5	116.9 (2)	C26—C30—H30E	109.5
C22—N5—H5	108.1	H30C—C30—H30E	109.5
Pd5—N5—H5	108.1	H30D—C30—H30E	109.5
C22—N5—H5A	108.1	C34—C31—C35	110.1 (3)
Pd5—N5—H5A	108.1	C34—C31—C32	109.8 (3)
H5—N5—H5A	107.3	C35—C31—C32	112.2 (3)
C27—N6—Pd6	117.2 (2)	C34—C31—S7	107.3 (3)
C27—N6—H6	108.0	C35—C31—S7	108.7 (3)
Pd6—N6—H6	108.0	C32—C31—S7	108.6 (3)
C27—N6—H6A	108.0	N7—C32—C33	110.7 (3)
Pd6—N6—H6A	108.0	N7—C32—C31	111.4 (3)
H6—N6—H6A	107.2	C33—C32—C31	112.4 (3)
C32—N7—Pd7	117.2 (2)	N7—C32—H32B	107.4
C32—N7—H7	108.0	C33—C32—H32B	107.4
Pd7—N7—H7	108.0	C31—C32—H32B	107.4
C32—N7—H7A	108.0	O14—C33—O13	122.0 (4)
Pd7—N7—H7A	108.0	O14—C33—C32	118.1 (4)
H7—N7—H7A	107.2	O13—C33—C32	119.8 (4)
C37—N8—Pd8	118.7 (2)	C31—C34—H34C	109.5
C37—N8—H8	107.6	C31—C34—H34D	109.5
Pd8—N8—H8	107.6	H34C—C34—H34D	109.5
C37—N8—H8A	107.6	C31—C34—H34E	109.5
Pd8—N8—H8A	107.6	H34C—C34—H34E	109.5
H8—N8—H8A	107.1	H34D—C34—H34E	109.5
C2—C1—C4	110.4 (3)	C31—C35—H35C	109.5
C2—C1—C5	113.6 (4)	C31—C35—H35D	109.5
C4—C1—C5	109.2 (4)	H35C—C35—H35D	109.5
C2—C1—S1	108.8 (3)	C31—C35—H35E	109.5
C4—C1—S1	106.4 (3)	H35C—C35—H35E	109.5
C5—C1—S1	108.2 (3)	H35D—C35—H35E	109.5
N1—C2—C1	110.1 (3)	C40—C36—C39	109.5 (3)
N1—C2—C3	110.5 (3)	C40—C36—C37	113.2 (3)
C1—C2—C3	112.4 (3)	C39—C36—C37	109.8 (4)
N1—C2—H2B	107.9	C40—C36—S8	109.0 (3)
C1—C2—H2B	107.9	C39—C36—S8	106.9 (3)
C3—C2—H2B	107.9	C37—C36—S8	108.3 (3)
O2—C3—O1	123.1 (4)	N8—C37—C38	110.4 (3)
O2—C3—C2	119.4 (4)	N8—C37—C36	110.9 (3)
O1—C3—C2	117.6 (4)	C38—C37—C36	113.3 (3)
C1—C4—H4C	109.5	N8—C37—H37B	107.3
C1—C4—H4D	109.5	C38—C37—H37B	107.3
H4C—C4—H4D	109.5	C36—C37—H37B	107.3
C1—C4—H4E	109.5	O16—C38—O15	122.7 (3)
H4C—C4—H4E	109.5	O16—C38—C37	118.2 (3)
H4D—C4—H4E	109.5	O15—C38—C37	119.1 (3)
C1—C5—H5C	109.5	C36—C39—H39C	109.5
C1—C5—H5D	109.5	C36—C39—H39D	109.5
H5C—C5—H5D	109.5	H39C—C39—H39D	109.5
C1—C5—H5E	109.5	C36—C39—H39E	109.5
H5C—C5—H5E	109.5	H39C—C39—H39E	109.5
H5D—C5—H5E	109.5	H39D—C39—H39E	109.5
C10—C6—C9	110.3 (4)	C36—C40—H40C	109.5
C10—C6—C7	112.6 (4)	C36—C40—H40D	109.5
C9—C6—C7	110.4 (4)	H40C—C40—H40D	109.5
C10—C6—S2	108.4 (3)	C36—C40—H40E	109.5
C9—C6—S2	106.8 (3)	H40C—C40—H40E	109.5
C7—C6—S2	108.2 (3)	H40D—C40—H40E	109.5
N2—C7—C8	111.4 (3)	H17F—O17—H17G	108 (3)
N2—C7—C6	109.7 (3)	H18F—O18—H18G	109 (3)
C8—C7—C6	113.4 (3)	H19F—O19—H19G	110 (3)
N2—C7—H7B	107.3	H20F—O20—H20G	108 (3)
C8—C7—H7B	107.3	H21F—O21—H21G	105 (3)
C6—C7—H7B	107.3	H22F—O22—H22G	107 (3)
O4—C8—O3	122.7 (4)	H23F—O23—H23G	107 (3)
O4—C8—C7	120.2 (4)	H24F—O24—H24G	108 (3)
O3—C8—C7	117.0 (4)	H25F—O25—H25G	105 (3)
C6—C9—H9C	109.5	H26F—O26—H26G	107 (3)
C6—C9—H9D	109.5	H27F—O27—H27G	109 (3)
H9C—C9—H9D	109.5	H28F—O28—H28G	116 (3)
C6—C9—H9E	109.5	H29F—O29—H29G	106 (3)
H9C—C9—H9E	109.5	H30F—O30—H30G	108 (3)
H9D—C9—H9E	109.5	H31F—O31—H31G	110 (3)
C6—C10—H10C	109.5	H32F—O32—H32G	101 (3)
C6—C10—H10D	109.5	H33F—O33—H33G	103 (3)
H10C—C10—H10D	109.5	H34F—O34—H34G	108 (3)
C6—C10—H10E	109.5	H35F—O35A—H35G	109 (3)
H10C—C10—H10E	109.5	H35H—O35B—H35I	113 (4)
H10D—C10—H10E	109.5	H36F—O36A—H36G	109 (3)
C14—C11—C12	111.5 (4)		
			
N1—Pd1—S1—C1	−23.21 (16)	Pd2—N2—C7—C8	−105.8 (3)
S4—Pd1—S1—C1	146.02 (13)	Pd2—N2—C7—C6	20.7 (4)
N1—Pd1—S1—Pd2	82.11 (9)	C10—C6—C7—N2	−165.2 (4)
S4—Pd1—S1—Pd2	−108.66 (4)	C9—C6—C7—N2	71.1 (4)
O1—Pd2—S1—C1	33.51 (16)	S2—C6—C7—N2	−45.5 (4)
S2—Pd2—S1—C1	−145.78 (14)	C10—C6—C7—C8	−39.9 (5)
O1—Pd2—S1—Pd1	−66.29 (9)	C9—C6—C7—C8	−163.7 (4)
S2—Pd2—S1—Pd1	114.43 (4)	S2—C6—C7—C8	79.8 (4)
N2—Pd2—S2—C6	−27.85 (19)	Pd3—O3—C8—O4	−126.5 (4)
S1—Pd2—S2—C6	144.23 (15)	Pd3—O3—C8—C7	55.4 (5)
N2—Pd2—S2—Pd3	76.25 (11)	N2—C7—C8—O4	−134.5 (4)
S1—Pd2—S2—Pd3	−111.67 (4)	C6—C7—C8—O4	101.2 (5)
N3—Pd3—S2—C6	−74.5 (5)	N2—C7—C8—O3	43.7 (5)
O3—Pd3—S2—C6	31.90 (17)	C6—C7—C8—O3	−80.7 (5)
S3—Pd3—S2—C6	−149.81 (14)	Pd3—S3—C11—C14	−77.6 (3)
N3—Pd3—S2—Pd2	−174.5 (4)	Pd4—S3—C11—C14	−176.7 (3)
O3—Pd3—S2—Pd2	−68.11 (10)	Pd3—S3—C11—C12	42.6 (3)
S3—Pd3—S2—Pd2	110.18 (4)	Pd4—S3—C11—C12	−56.4 (3)
N3—Pd3—S3—C11	−22.52 (17)	Pd3—S3—C11—C15	166.0 (3)
S2—Pd3—S3—C11	145.32 (14)	Pd4—S3—C11—C15	67.0 (3)
N3—Pd3—S3—Pd4	81.89 (11)	Pd3—N3—C12—C13	−99.3 (4)
S2—Pd3—S3—Pd4	−110.27 (4)	Pd3—N3—C12—C11	25.9 (4)
O5—Pd4—S3—C11	31.59 (17)	C14—C11—C12—N3	71.2 (4)
S4—Pd4—S3—C11	−147.27 (15)	C15—C11—C12—N3	−165.5 (3)
O5—Pd4—S3—Pd3	−68.27 (9)	S3—C11—C12—N3	−45.7 (4)
S4—Pd4—S3—Pd3	112.87 (4)	C14—C11—C12—C13	−165.2 (4)
N4—Pd4—S4—C16	−26.63 (15)	C15—C11—C12—C13	−41.9 (5)
S3—Pd4—S4—C16	144.88 (12)	S3—C11—C12—C13	77.9 (4)
N4—Pd4—S4—Pd1	76.65 (10)	Pd4—O5—C13—O6	−127.5 (4)
S3—Pd4—S4—Pd1	−111.85 (4)	Pd4—O5—C13—C12	52.3 (5)
O7—Pd1—S4—C16	29.29 (16)	N3—C12—C13—O6	−133.1 (4)
N1—Pd1—S4—C16	−79.6 (5)	C11—C12—C13—O6	103.3 (5)
S1—Pd1—S4—C16	−149.39 (14)	N3—C12—C13—O5	47.2 (5)
O7—Pd1—S4—Pd4	−71.63 (9)	C11—C12—C13—O5	−76.4 (5)
N1—Pd1—S4—Pd4	179.5 (5)	Pd4—S4—C16—C20	166.7 (2)
S1—Pd1—S4—Pd4	109.69 (4)	Pd1—S4—C16—C20	62.3 (3)
N5—Pd5—S5—C21	−23.67 (15)	Pd4—S4—C16—C19	−74.0 (3)
S8—Pd5—S5—C21	146.60 (12)	Pd1—S4—C16—C19	−178.4 (3)
N5—Pd5—S5—Pd6	80.95 (9)	Pd4—S4—C16—C17	44.2 (3)
S8—Pd5—S5—Pd6	−108.78 (4)	Pd1—S4—C16—C17	−60.3 (3)
N6—Pd6—S5—C21	−70.4 (5)	Pd4—N4—C17—C18	−108.1 (3)
O9—Pd6—S5—C21	35.97 (16)	Pd4—N4—C17—C16	18.9 (4)
S6—Pd6—S5—C21	−143.07 (14)	C20—C16—C17—N4	−161.8 (3)
N6—Pd6—S5—Pd5	−171.1 (5)	C19—C16—C17—N4	73.9 (4)
O9—Pd6—S5—Pd5	−64.77 (9)	S4—C16—C17—N4	−42.5 (4)
S6—Pd6—S5—Pd5	116.19 (4)	C20—C16—C17—C18	−37.2 (5)
N6—Pd6—S6—C26	−27.51 (18)	C19—C16—C17—C18	−161.4 (3)
S5—Pd6—S6—C26	142.24 (14)	S4—C16—C17—C18	82.1 (3)
N6—Pd6—S6—Pd7	77.14 (11)	Pd1—O7—C18—O8	−149.9 (3)
S5—Pd6—S6—Pd7	−113.11 (4)	Pd1—O7—C18—C17	33.2 (5)
O11—Pd7—S6—C26	28.82 (17)	N4—C17—C18—O8	−116.7 (4)
S7—Pd7—S6—C26	−151.02 (15)	C16—C17—C18—O8	117.9 (4)
O11—Pd7—S6—Pd6	−70.75 (10)	N4—C17—C18—O7	60.4 (5)
S7—Pd7—S6—Pd6	109.40 (4)	C16—C17—C18—O7	−65.0 (5)
N7—Pd7—S7—C31	−22.31 (16)	Pd5—S5—C21—C24	−74.9 (3)
S6—Pd7—S7—C31	148.75 (14)	Pd6—S5—C21—C24	−176.4 (3)
N7—Pd7—S7—Pd8	81.70 (10)	Pd5—S5—C21—C22	44.1 (3)
S6—Pd7—S7—Pd8	−107.25 (4)	Pd6—S5—C21—C22	−57.4 (3)
O13—Pd8—S7—C31	30.71 (16)	Pd5—S5—C21—C25	166.4 (3)
S8—Pd8—S7—C31	−147.56 (13)	Pd6—S5—C21—C25	64.9 (3)
O13—Pd8—S7—Pd7	−69.71 (9)	Pd5—N5—C22—C23	−98.2 (3)
S8—Pd8—S7—Pd7	112.02 (4)	Pd5—N5—C22—C21	26.2 (4)
N8—Pd8—S8—C36	−26.71 (15)	C24—C21—C22—N5	69.7 (4)
S7—Pd8—S8—C36	144.26 (12)	C25—C21—C22—N5	−165.8 (3)
N8—Pd8—S8—Pd5	77.31 (10)	S5—C21—C22—N5	−46.9 (4)
S7—Pd8—S8—Pd5	−111.73 (4)	C24—C21—C22—C23	−165.7 (3)
O15—Pd5—S8—C36	27.72 (15)	C25—C21—C22—C23	−41.3 (4)
N5—Pd5—S8—C36	−85.4 (6)	S5—C21—C22—C23	77.6 (4)
S5—Pd5—S8—C36	−151.84 (13)	Pd6—O9—C23—O10	−117.2 (4)
O15—Pd5—S8—Pd8	−72.85 (9)	Pd6—O9—C23—C22	63.4 (4)
N5—Pd5—S8—Pd8	174.1 (5)	N5—C22—C23—O10	−138.9 (4)
S5—Pd5—S8—Pd8	107.59 (4)	C21—C22—C23—O10	97.1 (5)
N2—Pd2—O1—C3	132.9 (3)	N5—C22—C23—O9	40.6 (5)
S1—Pd2—O1—C3	−39.1 (3)	C21—C22—C23—O9	−83.4 (4)
N3—Pd3—O3—C8	133.2 (4)	Pd6—S6—C26—C30	169.5 (3)
S2—Pd3—O3—C8	−34.8 (3)	Pd7—S6—C26—C30	68.4 (3)
N4—Pd4—O5—C13	139.1 (3)	Pd6—S6—C26—C27	46.4 (3)
S3—Pd4—O5—C13	−32.4 (3)	Pd7—S6—C26—C27	−54.6 (3)
N1—Pd1—O7—C18	149.4 (3)	Pd6—S6—C26—C29	−72.0 (3)
S4—Pd1—O7—C18	−19.7 (3)	Pd7—S6—C26—C29	−173.0 (3)
N6—Pd6—O9—C23	128.3 (3)	Pd6—N6—C27—C28	−106.6 (3)
S5—Pd6—O9—C23	−41.4 (3)	Pd6—N6—C27—C26	20.1 (4)
N7—Pd7—O11—C28	143.1 (3)	C30—C26—C27—N6	−165.3 (4)
S6—Pd7—O11—C28	−27.9 (3)	C29—C26—C27—N6	71.4 (4)
N8—Pd8—O13—C33	145.6 (4)	S6—C26—C27—N6	−44.9 (4)
S7—Pd8—O13—C33	−25.3 (3)	C30—C26—C27—C28	−42.0 (5)
N5—Pd5—O15—C38	150.5 (3)	C29—C26—C27—C28	−165.3 (4)
S8—Pd5—O15—C38	−19.8 (3)	S6—C26—C27—C28	78.4 (4)
O7—Pd1—N1—C2	−178.3 (3)	Pd7—O11—C28—O12	−135.9 (4)
S1—Pd1—N1—C2	1.9 (2)	Pd7—O11—C28—C27	46.5 (5)
S4—Pd1—N1—C2	−68.3 (6)	N6—C27—C28—O12	−127.5 (5)
O1—Pd2—N2—C7	−171.9 (3)	C26—C27—C28—O12	108.3 (5)
S2—Pd2—N2—C7	8.5 (3)	N6—C27—C28—O11	50.2 (5)
O3—Pd3—N3—C12	178.3 (3)	C26—C27—C28—O11	−74.0 (5)
S3—Pd3—N3—C12	1.8 (3)	Pd7—S7—C31—C34	−77.1 (3)
S2—Pd3—N3—C12	−73.8 (6)	Pd8—S7—C31—C34	−178.8 (2)
O5—Pd4—N4—C17	−171.7 (3)	Pd7—S7—C31—C35	163.9 (3)
S4—Pd4—N4—C17	8.5 (2)	Pd8—S7—C31—C35	62.2 (3)
O15—Pd5—N5—C22	−178.4 (3)	Pd7—S7—C31—C32	41.5 (3)
S5—Pd5—N5—C22	2.5 (2)	Pd8—S7—C31—C32	−60.2 (3)
S8—Pd5—N5—C22	−64.3 (6)	Pd7—N7—C32—C33	−100.4 (3)
O9—Pd6—N6—C27	−171.9 (3)	Pd7—N7—C32—C31	25.4 (4)
S6—Pd6—N6—C27	8.6 (3)	C34—C31—C32—N7	72.0 (4)
S5—Pd6—N6—C27	−64.6 (6)	C35—C31—C32—N7	−165.2 (3)
O11—Pd7—N7—C32	−179.3 (3)	S7—C31—C32—N7	−45.0 (4)
S7—Pd7—N7—C32	2.0 (3)	C34—C31—C32—C33	−163.1 (3)
O13—Pd8—N8—C37	−171.1 (3)	C35—C31—C32—C33	−40.3 (4)
S8—Pd8—N8—C37	8.7 (2)	S7—C31—C32—C33	79.9 (3)
Pd1—S1—C1—C2	44.4 (3)	Pd8—O13—C33—O14	−142.1 (4)
Pd2—S1—C1—C2	−55.3 (3)	Pd8—O13—C33—C32	40.9 (5)
Pd1—S1—C1—C4	−74.6 (3)	N7—C32—C33—O14	−119.8 (4)
Pd2—S1—C1—C4	−174.3 (3)	C31—C32—C33—O14	115.0 (4)
Pd1—S1—C1—C5	168.2 (3)	N7—C32—C33—O13	57.4 (5)
Pd2—S1—C1—C5	68.5 (3)	C31—C32—C33—O13	−67.9 (5)
Pd1—N1—C2—C1	26.9 (4)	Pd8—S8—C36—C40	167.9 (2)
Pd1—N1—C2—C3	−97.9 (3)	Pd5—S8—C36—C40	64.6 (3)
C4—C1—C2—N1	68.7 (4)	Pd8—S8—C36—C39	−73.9 (3)
C5—C1—C2—N1	−168.2 (3)	Pd5—S8—C36—C39	−177.1 (3)
S1—C1—C2—N1	−47.7 (4)	Pd8—S8—C36—C37	44.3 (3)
C4—C1—C2—C3	−167.6 (4)	Pd5—S8—C36—C37	−58.9 (3)
C5—C1—C2—C3	−44.5 (5)	Pd8—N8—C37—C38	−107.5 (3)
S1—C1—C2—C3	76.0 (4)	Pd8—N8—C37—C36	18.9 (4)
Pd2—O1—C3—O2	−118.8 (4)	C40—C36—C37—N8	−163.6 (3)
Pd2—O1—C3—C2	60.9 (4)	C39—C36—C37—N8	73.8 (4)
N1—C2—C3—O2	−137.9 (4)	S8—C36—C37—N8	−42.6 (4)
C1—C2—C3—O2	98.6 (5)	C40—C36—C37—C38	−38.7 (5)
N1—C2—C3—O1	42.4 (5)	C39—C36—C37—C38	−161.4 (3)
C1—C2—C3—O1	−81.1 (5)	S8—C36—C37—C38	82.3 (3)
Pd2—S2—C6—C10	169.6 (3)	Pd5—O15—C38—O16	−146.6 (3)
Pd3—S2—C6—C10	67.1 (3)	Pd5—O15—C38—C37	35.6 (5)
Pd2—S2—C6—C9	−71.5 (3)	N8—C37—C38—O16	−120.8 (4)
Pd3—S2—C6—C9	−174.1 (3)	C36—C37—C38—O16	114.1 (4)
Pd2—S2—C6—C7	47.3 (3)	N8—C37—C38—O15	57.1 (5)
Pd3—S2—C6—C7	−55.3 (3)	C36—C37—C38—O15	−68.0 (4)

### Hydrogen-bond geometry (Å, º)

**Table:**

*D*—H···*A*	*D*—H	H···*A*	*D*···*A*	*D*—H···*A*
N1—H1···O26^i^	0.92	2.06	2.963 (5)	169
N1—H1*A*···O15^ii^	0.92	2.21	3.109 (4)	166
N2—H2···O27	0.92	2.35	3.139 (5)	143
N2—H2*A*···O13^ii^	0.92	2.11	2.928 (5)	148
N3—H3···O35*B*	0.92	2.01	2.889 (8)	160
N3—H3···O36*A*	0.92	2.23	3.045 (8)	147
N3—H3*A*···O11^ii^	0.92	2.37	3.278 (5)	167
N4—H4···O29^iii^	0.92	1.98	2.886 (5)	170
N4—H4*A*···O9^ii^	0.92	2.04	2.917 (4)	160
N5—H5···O18^i^	0.92	2.09	3.008 (5)	172
N5—H5*A*···O7^iv^	0.92	2.19	3.094 (4)	167
N6—H6···O27^v^	0.92	2.44	3.183 (5)	138
N6—H6···O28	0.92	2.48	3.326 (5)	153
N6—H6*A*···O5^iv^	0.92	2.21	3.041 (5)	151
N7—H7···O23	0.92	2.03	2.938 (5)	170
N7—H7*A*···O3^iv^	0.92	2.16	3.066 (5)	169
N8—H8···O28^vi^	0.92	1.98	2.896 (5)	171
N8—H8*A*···O1^iv^	0.92	2.06	2.930 (4)	157
O17—H17*F*···O26^iv^	0.85 (2)	1.96 (2)	2.798 (5)	167 (6)
O17—H17*G*···O16^vii^	0.86 (2)	1.93 (2)	2.775 (4)	170 (5)
O18—H18*F*···O17	0.84 (2)	2.00 (3)	2.818 (5)	165 (6)
O18—H18*G*···O20	0.85 (2)	1.98 (2)	2.814 (5)	165 (5)
O19—H19*F*···O8^vii^	0.85 (2)	2.05 (2)	2.892 (5)	168 (5)
O19—H19*G*···O2^viii^	0.85 (2)	1.94 (3)	2.760 (5)	160 (6)
O20—H20*F*···O21	0.85 (2)	1.88 (2)	2.709 (5)	167 (6)
O20—H20*G*···O8^ix^	0.86 (2)	1.94 (3)	2.742 (4)	156 (5)
O21—H21*F*···O22	0.86 (2)	1.97 (3)	2.805 (6)	162 (5)
O21—H21*G*···O30	0.87 (2)	2.00 (2)	2.857 (6)	169 (6)
O22—H22*F*···O10^vii^	0.85 (2)	1.95 (2)	2.802 (5)	174 (6)
O22—H22*G*···O16^viii^	0.86 (2)	2.21 (4)	2.915 (5)	139 (6)
O23—H23*F*···O20	0.85 (2)	1.96 (2)	2.796 (5)	168 (5)
O23—H23*G*···O25^iv^	0.85 (2)	1.96 (2)	2.782 (5)	162 (5)
O24—H24*F*···O19^vi^	0.85 (2)	1.97 (2)	2.814 (5)	177 (6)
O24—H24*G*···O32	0.85 (2)	1.90 (2)	2.736 (6)	168 (6)
O25—H25*F*···O24^iii^	0.87 (2)	1.91 (2)	2.770 (6)	170 (6)
O25—H25*G*···O12^ii^	0.85 (2)	1.97 (3)	2.788 (5)	159 (5)
O26—H26*F*···O23^ii^	0.86 (2)	1.95 (2)	2.791 (5)	167 (6)
O26—H26*G*···O31	0.85 (2)	1.95 (2)	2.788 (6)	169 (6)
O27—H27*F*···O6^vi^	0.85 (2)	1.88 (3)	2.687 (5)	158 (5)
O27—H27*G*···O14^ii^	0.84 (2)	1.81 (2)	2.650 (5)	175 (5)
O28—H28*F*···O10	0.82 (2)	2.02 (2)	2.810 (5)	161 (5)
O28—H28*G*···O27^v^	0.82 (2)	2.00 (3)	2.797 (5)	164 (5)
O29—H29*F*···O2	0.87 (2)	1.98 (2)	2.834 (5)	168 (6)
O29—H29*G*···O27	0.85 (2)	1.98 (2)	2.802 (5)	160 (5)
O30—H30*F*···O12	0.86 (2)	2.28 (5)	2.911 (7)	130 (6)
O30—H30*G*···O19^iv^	0.86 (2)	1.95 (3)	2.791 (5)	165 (6)
O31—H31*F*···O33^ii^	0.84 (2)	2.17 (3)	2.946 (8)	154 (7)
O31—H31*G*···O4	0.86 (2)	2.08 (2)	2.902 (6)	161 (5)
O32—H32*F*···O34	0.92 (2)	2.07 (2)	2.823 (7)	139 (3)
O32—H32*G*···O31	0.86 (2)	1.98 (2)	2.837 (7)	178 (9)
O33—H33*F*···O17	0.88 (2)	2.11 (4)	2.877 (6)	146 (7)
O33—H33*G*···O30^vi^	0.87 (2)	1.90 (2)	2.766 (6)	170 (7)
O34—H34*F*···O18	0.85 (2)	2.12 (4)	2.906 (6)	154 (8)
O34—H34*G*···O6^vi^	0.86 (2)	2.02 (3)	2.815 (6)	154 (6)
O35*A*—H35*F*···O25	0.85 (2)	2.14 (3)	2.986 (9)	174 (13)
O35*A*—H35*G*···O4	0.85 (2)	2.05 (5)	2.848 (9)	157 (12)
O35*B*—H35*H*···O4	0.83 (2)	2.45 (8)	3.135 (9)	141 (10)
O35*B*—H35*I*···O25	0.84 (2)	2.03 (5)	2.802 (10)	152 (9)
O36*A*—H36*F*···O22	0.85 (2)	2.14 (5)	2.949 (9)	159 (12)
O36*A*—H36*G*···O35*A*	0.85 (2)	1.93 (5)	2.746 (12)	158 (12)

Symmetry codes: (i) *x*, *y*, *z*−1; (ii) *x*, *y*−1, *z*; (iii) *x*+1, *y*, *z*; (iv) *x*, *y*+1, *z*; (v) *x*+1, *y*+1, *z*; (vi) *x*−1, *y*, *z*; (vii) *x*, *y*, *z*+1; (viii) *x*+1, *y*, *z*+1; (ix) *x*, *y*+1, *z*+1.
